# Supplementary material for: Parental experiences orchestrate locust egg hatching synchrony by regulating nuclear export of precursor miRNA
Source: Nat Commun. 2024 May 21;15:4328. doi: 10.1038/s41467-024-48658-7 (PMC11109280; doi:10.1038/s41467-024-48658-7)
Supplement: Supplementary file 1 — Supplementary Information [file 41467_2024_48658_MOESM1_ESM.pdf]

Supplementary Information for

Parental experiences orchestrate the locust egg hatching synchrony of offspring by regulating nuclear export of precursor miRNA

Ya′nan Zhu, Jing He *et al*.

*Corresponding author: lkang@ioz.ac.cn

This PDF file includes:

Supplementary Fig. 1. Schematic diagram of precursor miR-276 (pre-miR-276) probe designed for *in situ* hybridization.

Supplementary Fig. 2. Negative control (NC) for FISH assay in ovarioles by using NC probe.

Supplementary Fig. 3. Uncropped gel with input of RNA pull-down assay.

Supplementary Fig. 4. The efficiency of RNAi determined by qPCR.

Supplementary Fig. 5. The gel images of U6 snRNA and 18S rRNA.

Supplementary Fig. 6. The expression levels of mRNA (a) and protein (b) of Paired (Prd) in the terminal oocytes of gregarious (G) and solitarious (S) locusts.

Supplementary Fig. 7. Electrophoretic mobility shift assay (EMSA) of the binding of nuclear protein in the terminal oocytes of locusts with the probe for FOXN1.

Supplementary Fig. 8. Confirmation of prokaryotically expressed PTBP1 by Western blotting.

Supplementary Fig. 9. The nucleotide sequence of wild type (WT) and knockout (KO) of XPO5.

Supplementary Fig. 10. Determination of the binding of selected eight pre-miRNAs with PTBP1 and XPO5 by RIP assays in terminal oocytes.

Supplementary Fig. 11. Expression of miRNAs produced from the plasmids with single or double mutation sites.

Supplementary Fig. 12. The sequences of pre-miR-305 and pre-miR-34.

Supplementary Fig. 13. Frequency distribution histogram of the egg-hatching time.

Supplementary Fig. 14. The transmission of expression patterns of PTBP1, miR-276, and BRM from female terminal oocytes to progeny eggs.

Supplementary Fig. 15. The full-size gel scans corresponding to the main figures.

Supplementary Fig. 16. Validation of the specificity of the antibody against locust PTBP1, XPO5 and Prd protein.

Supplementary Fig. 17. The full-size Western blot scans corresponding to the main figures.

Supplementary Fig. 18. The full-size Western blot scans corresponding to Supplementary Figures.

Supplementary Table 1. The list of proteins that specifically pulled down by pre-miR-276.

Supplementary Table 2. The analysis results of hatching synchrony within egg-pods.

Supplementary Figures

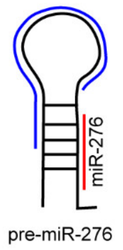

Supplementary Fig. 1. Schematic diagram of precursor miR-276 (pre-miR-276) probe designed for *in situ* hybridization. The blue curve and red line indicate the location of the probe and the location of miR-276, respectively.

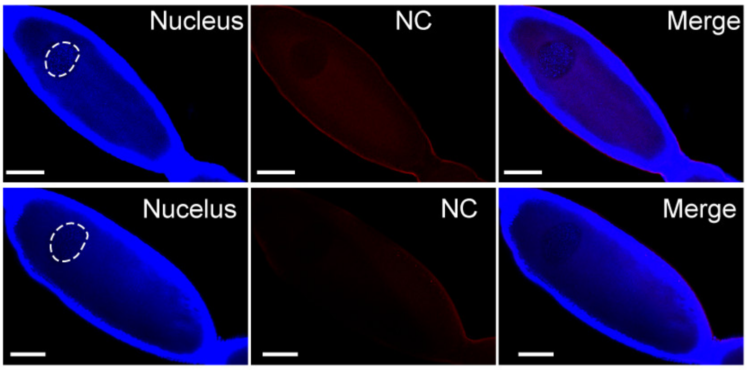

Supplementary Fig. 2. Negative control (NC) for FISH assay in ovarioles by using NC probe. The NC probe showed no signals in terminal oocytes. Results of two biologically independent locusts are shown. The experiment was performed one time. The nucleus of the terminal oocytes is delineated by a white dotted line circle. Scale bar: 100 μm.

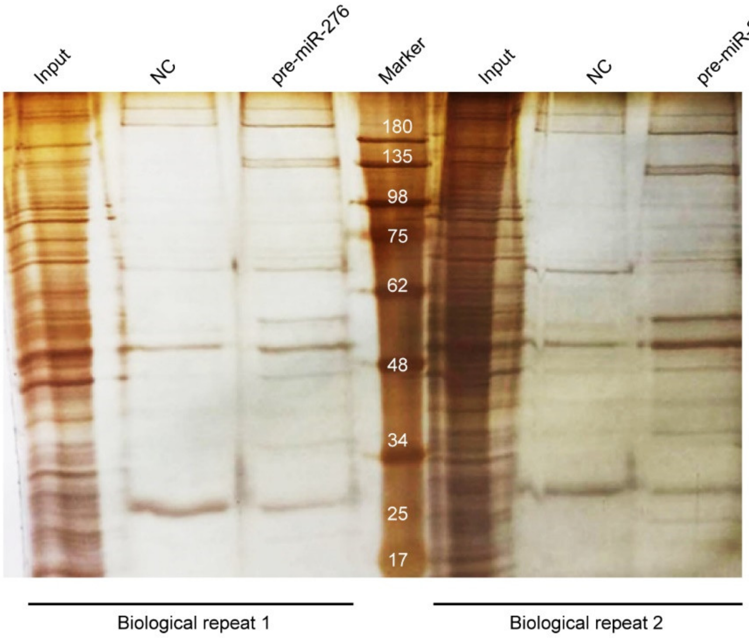

Supplementary Fig. 3. Uncropped gel with input of RNA pull-down assay. Results of two biologically independent locusts are shown. The experiment was performed one time. NC: negative control.

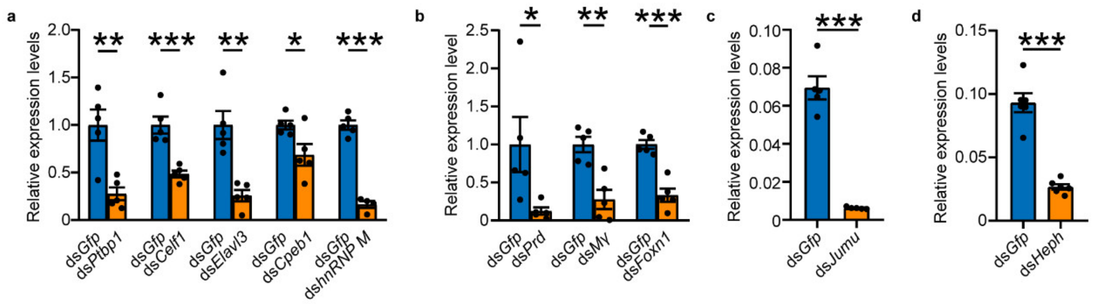

Supplementary Fig. 4. The efficiency of RNAi determined by qPCR. (**a**) The RNAi efficiency of the candidate genes that may participate in nuclear export of pre-miR-276 in terminal oocytes of locusts (*n* =5 biologically independent locusts). (**b**) The RNAi efficiency of the potential TFs of *Ptbp1* in terminal oocytes of locusts (*n* =5 biologically independent locusts). (**c-d**) The RNAi efficiency of *Drosophila Jumu* and *Heph* in S2 cells (*n* = 5 and 6 biologically independent cell samples for *Jumu* and *Heph* group, respectively. Each cell sample was from one well of a 48-well plate). Student's *t* test (two-tailed) was used for two-group comparisons. The data are shown as mean ± SEM. * $P < 0.05$, ** $P < 0.01$, *** $P < 0.001$. Source data and details of the statistical results are provided as a Source Data file.

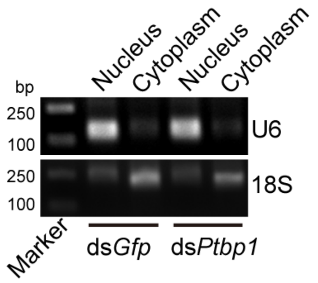

Supplementary Fig. 5. The gel images of U6 snRNA and 18S rRNA. U6 snRNA and 18S rRNA were used as nuclear and cytoplasmic markers of terminal oocytes after female locusts injecting ds*Gfp* or ds*Ptbp1*. The experiment was performed one time and the results of amplifications were validated by sequencing. Supplementary Fig. 15d shows the original images.

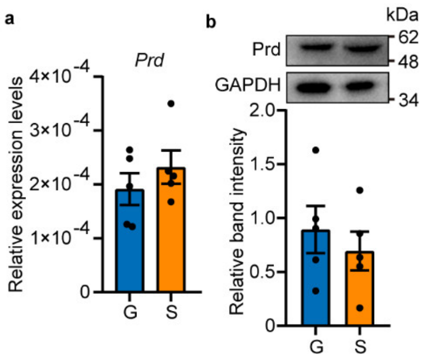

Supplementary Fig. 6. The expression levels of mRNA (a) and protein (b) of Paired (Prd) in the terminal oocytes of gregarious (G) and solitarious (S) locusts (*n* = 5 biologically independent locusts)**.** Supplementary Fig. 18a shows the original images of Western blots. Student's *t* test (two-tailed) was used for two-group comparisons. The data are shown as mean ± SEM. Source data and details of the statistical results are provided as a Source Data file.

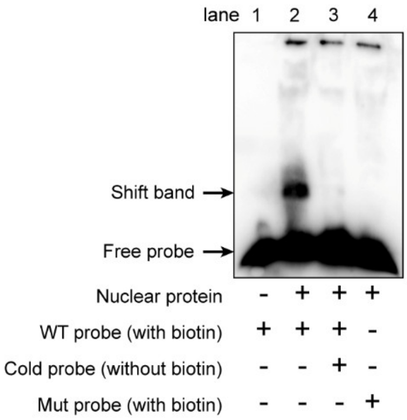

Supplementary Fig. 7. Electrophoretic mobility shift assay (EMSA) of the binding of nuclear protein in the terminal oocytes of locusts with the probe for FOXN1. Both the wild type (WT) and mutant (Mut) probe were labeled with biotin, while the cold probe was unlabeled.

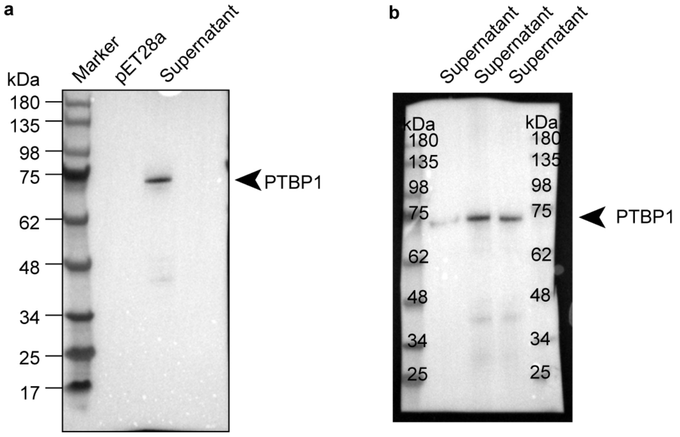

Supplementary Fig. 8. Confirmation of prokaryotically expressed PTBP1 by Western blotting. The supernatant from the induction of PTBP1 expression in *E.coli* was tested using PTBP1 antibodies, with the empty pET28a vector serving as the negative control. The arrowhead indicates the band of PTBP1. The detection of prokaryotically expressed PTBP1 by Western blotting was repeated by three times, and (a) and (b) indicate four technological repeats with similar results.

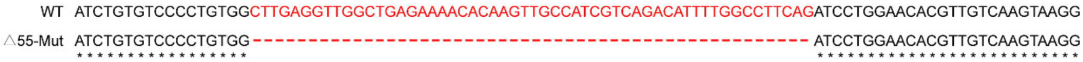

Supplementary Fig. 9. The nucleotide sequence of wild type (WT) and knockout (KO) of XPO5. The 55 nucleotides that were completely deleted in the mutated cells are highlighted in red. WT: wild type sequences; Δ55-Mut: mutant sequences with a deletion of 55 nucleotides.

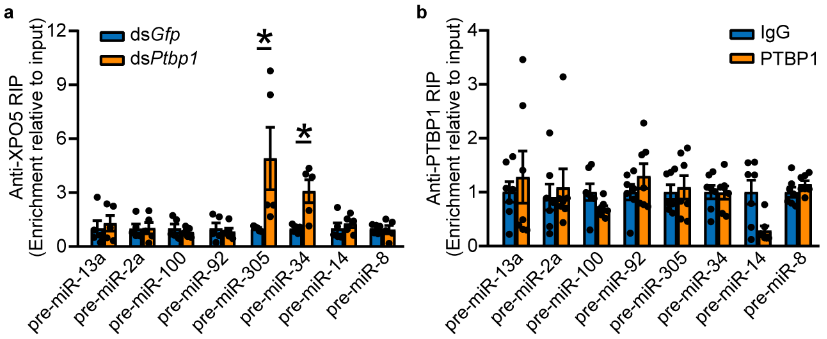

Supplementary Fig. 10. Determination of the binding of selected eight pre-miRNAs with PTBP1 and XPO5 by RIP assays in terminal oocytes. (a) RIP assay to determine the abundances of the eight selected pre-miRNAs in immunoprecipitates of XPO5 in female locust terminal oocytes after *Ptbp1* knockdown (n = 5 biologically independent locusts). (**b**) qPCR analysis of the eight selected pre-miRNAs in PTBP1 immunoprecipitates from female locust terminal oocyte lysates (n = 7 biologically independent locusts). Mann–Whitney U test (two-tailed) was applied for two-group comparisons of pre-miR-13a, pre-miR-2a and pre-miR-14 in (b), and Student's t test (two-tailed) was used for other two-group comparisons. The data are shown as mean ± SEM. * $P < 0.05$. Source data and details of the statistical results are provided as a Source Data file.

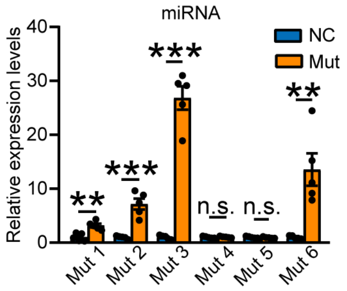

Supplementary Fig. 11. Expression of miRNAs produced from the plasmids with single or double mutation sites. *n* = 5 biologically independent locusts. Student's *t* test (two-tailed) was used for two-group comparisons. NC: negative control (empty vector); Mut: mutant vector. The data are shown as mean ± SEM. ** $P < 0.01$, *** $P < 0.001$, n.s. no significant difference. Source data and details of the statistical results are provided as a Source Data file.

pre-miR-305	GCCUCAUGUUCAUUGUACUUCAUCAGGUGCUCUGGUGUGACGGAACCCGGCACCUGUUGGAGUGCAAUUGAUAGGAAGG
pre-miR-34	GCCGCAGUGGCAGUGUGGUUAGCUGGUUGUGUGGAAUUUGCUCACUGCCACUAUCCGCACUGCCUUGCGACC

Supplementary Fig. 12. The sequences of pre-miR-305 and pre-miR-34.

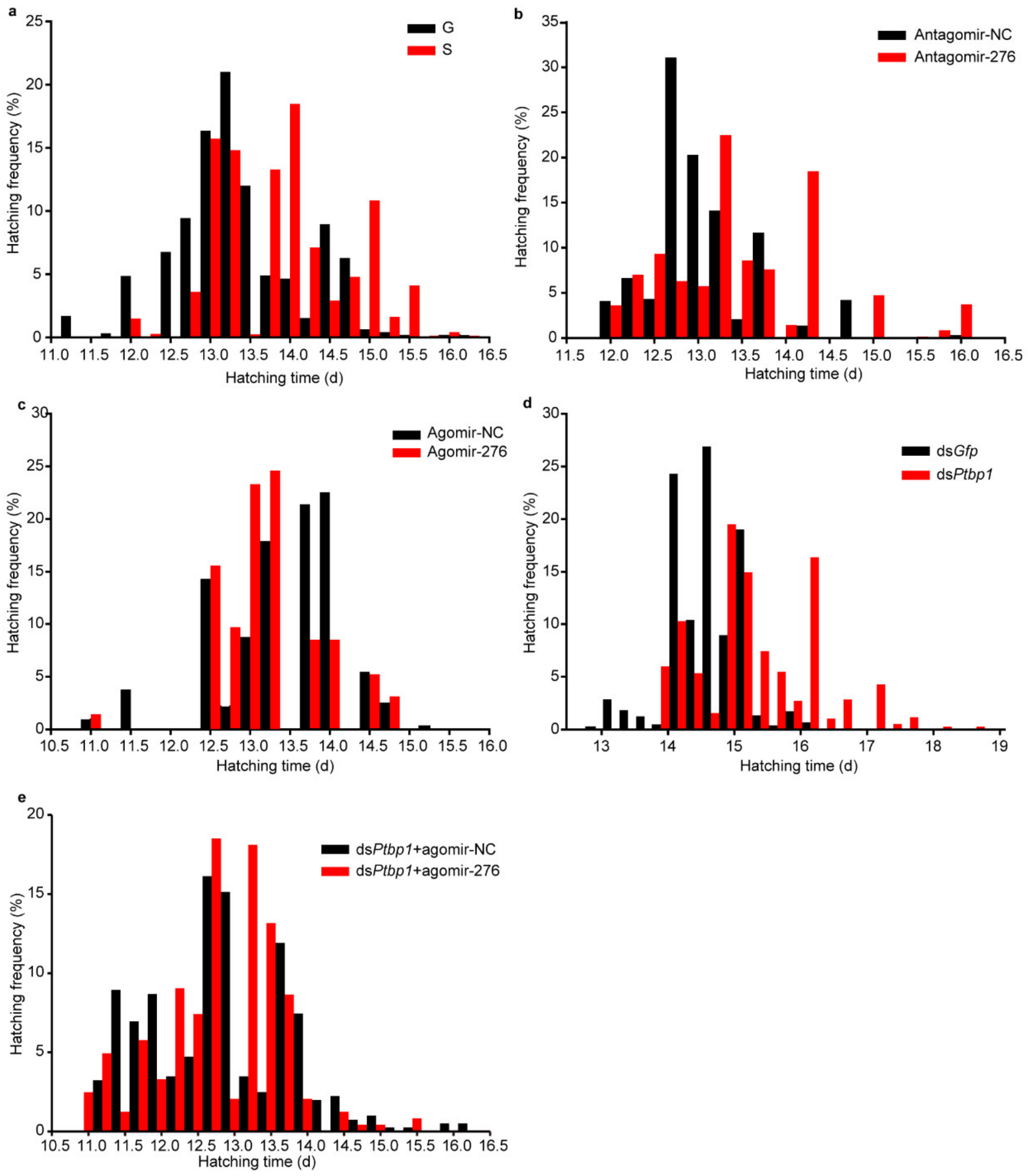

Supplementary Fig. 13. Frequency distribution histogram of the egg-hatching time. (a) The frequency of hatching time in eggs of gregarious and solitarious females [n = 1,850 biologically independent eggs for gregarious (G) and solitarious (S) locusts; Levene's test, P = 1.38E-06; F-test, P = 0.010]. (b) The effects of inhibition of miR-276 in gregarious females on hatching time of progeny eggs (n = 1,320 biologically independent eggs; Levene's test, P = 7.09E-58; F-test, P < 0.0001). (c) Effects of overexpression of miR-276 in solitarious females on hatching time of progeny eggs (n = 978 biologically independent eggs, Levene's test, P = 3.67E-14; F-test, P < 0.0001). (d) Effects of *Ptbp1* knockdown in gregarious locusts on hatching time of progeny eggs (n = 1,041 and 769 biologically independent eggs for ds*Gfp* and for ds*Ptbp1*, respectively; Levene's test, P = 1.54E-58; F-test, P < 0.0001). (e) The effects of

overexpression of miR-276 in hatching time of eggs from gregarious females that were pre-treated by *Ptbp1* knockdown (n = 403 and 243 biologically independent eggs for agomir-NC and agomir-276, respectively; Levene's test, $P = 0.007$; F-test, $P = 0.003$). Antagomir-276 was a chemically modified single-strand RNA complemented with miR-276, and was used to inhibit miR-276 in locusts; agomir-276 was a chemically modified double-strand miR-276 mimic, and was used to overexpression of miR-276. NC was negative control for antagomir-276 or agomir-276. *Gfp* was used as the negative control for *Ptbp1* knockdown. Levene's test (one-tailed) and F-test (one-tailed) were used to analyze the variation of the egg hatching time. Source data and details of the statistical results are provided as a Source Data file.

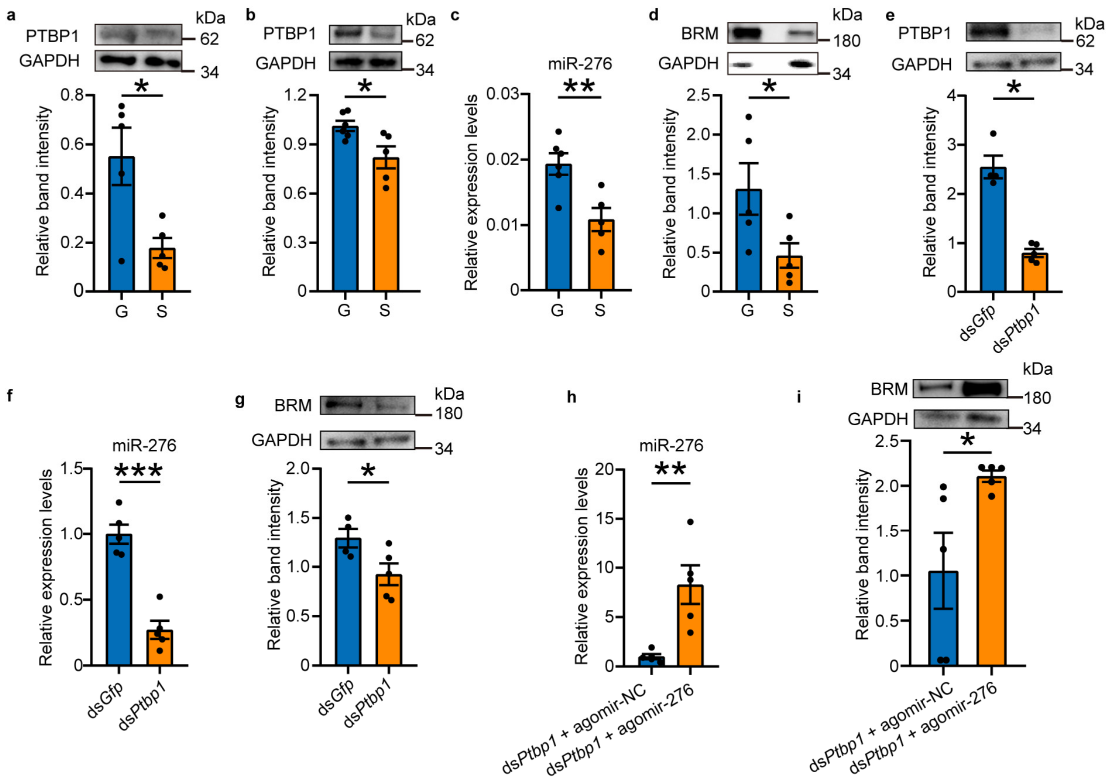

Supplementary Fig. 14. The transmission of expression patterns of PTBP1, miR-276, and BRM from female terminal oocytes to progeny eggs. (**a-b**) The abundance of PTBP1 determined by immunoblotting in the terminal oocytes (a) and eggs (b) of gregarious and solitarious locusts (for terminal oocytes: n = 5 biologically independent locusts; for eggs: n = 6 biologically independent eggs for G, n = 5 biologically independent eggs for S). Supplementary Fig. 18b and 18c show the original images of Western blots. (**c-d**) The abundance of miR-276 (c) and BRM (d) in eggs of gregarious locusts and solitarious locusts (for miR-276: n = 6 biologically independent eggs for G, n = 5 biologically independent eggs for S; for BRM: n = 5 biologically independent eggs). Supplementary Fig. 18d shows the original images of Western blots. (**e-g**) The abundance of PTBP1 (e), miR-276 (f), and BRM (g) in eggs of gregarious locusts injected with ds*Gfp* or ds*Ptbp1* (for PTBP1: n = 4 biologically independent eggs for ds*Gfp*, n = 5 biologically independent eggs for ds*Ptbp1*; for miR-276: n = 5 biologically independent eggs; for BRM: n = 4 biologically independent eggs for ds*Gfp*, n = 5 biologically independent eggs for ds*Ptbp1*). Supplementary Fig. 18e and 18f show the original image of Western blots. (**h-i**) The abundance of miR-276 (h) and BRM (i) in eggs of gregarious locusts injected with agomir-276 or agomir-NC after knocking down *Ptbp1* (for miR-276: n = 5

biologically independent eggs; for BRM: n =5 biologically independent eggs). Supplementary Fig. 18g shows the original images of Western blots. G: gregarious locusts; S: solitarious locusts; NC: negative control for miR-276. *Gfp* was used as the negative control for *Ptbp1* knockdown. Mann–Whitney U test (two-tailed) was applied for two-group comparisons in (e), and Student's t test (two-tailed) was applied for other two-group comparisons. The data are shown as mean ± SEM. * $P < 0.05$, ** $P < 0.01$, *** $P < 0.001$. Source data and details of the statistical results are provided as a Source Data file.

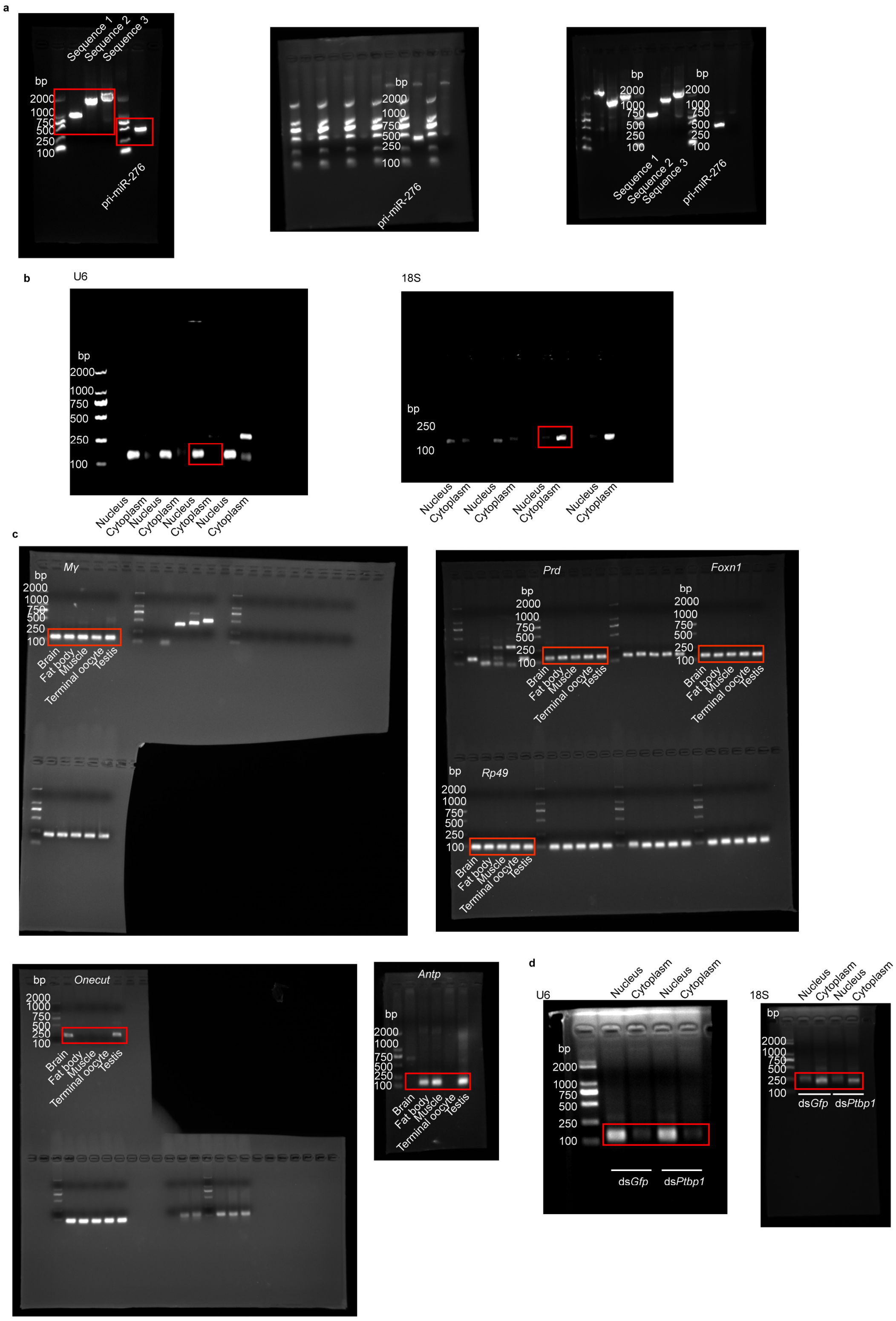

Supplementary Fig. 15. The full-size gel scans corresponding to the main figures. (a) The uncropped gel scans of amplification of promoter in Fig. 3c and primary miR-276 (pri-miR-276) in Fig. 1c. The experiments were performed three times with similar results for pri-miR-276, and twice with the similar results for promoter. (b) The uncropped gel scans of U6 snRNA

and 18S in Fig. 1h. The results performed by three biologically independent locusts are shown in the images. (c) The uncropped gel scans of amplification of candidate TFs and *Rp49* in different tissues in Fig. 3f. *Rp49* were used as endogenous controls for genes. (d) The uncropped gel scans of U6 snRNA and 18S in Supplementary Fig. 5. The bands that were cropped and presented in the main figures are indicated in red frames. The bands excluded from the red frames in c represent other samples that are unrelated to this study.

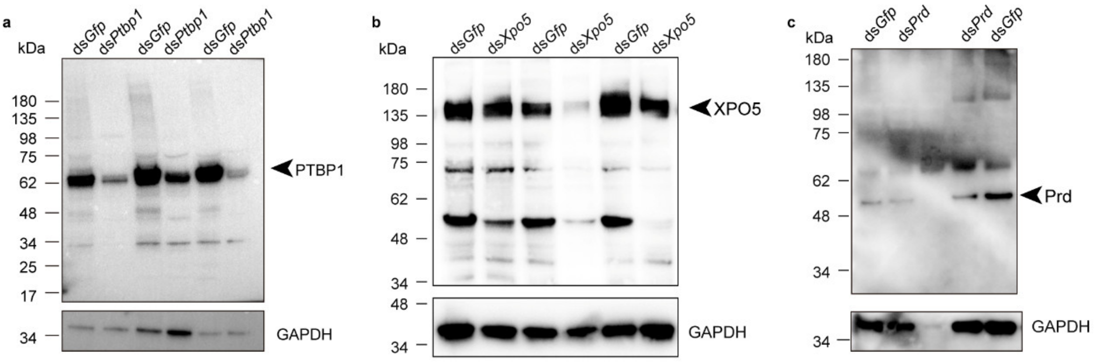

Supplementary Fig. 16. Validation of the specificity of the antibody against locust PTBP1, XPO5 and Prd protein. Immunoblotting of PTBP1 (a), XPO5 (b) or Prd (c) after knocking down of *Ptbp1*, *Xpo5* or *Prd* in terminal oocytes of locusts, respectively. n = 3 biologically independent locusts for (a) and (b), and n = 2 biologically independent locusts for (c). The arrowhead indicates the band of PTBP1, XPO5 or Prd. Injection of dsRNAs against *Gfp* was used as the negative control. GAPDH on the same gel was used as internal control.

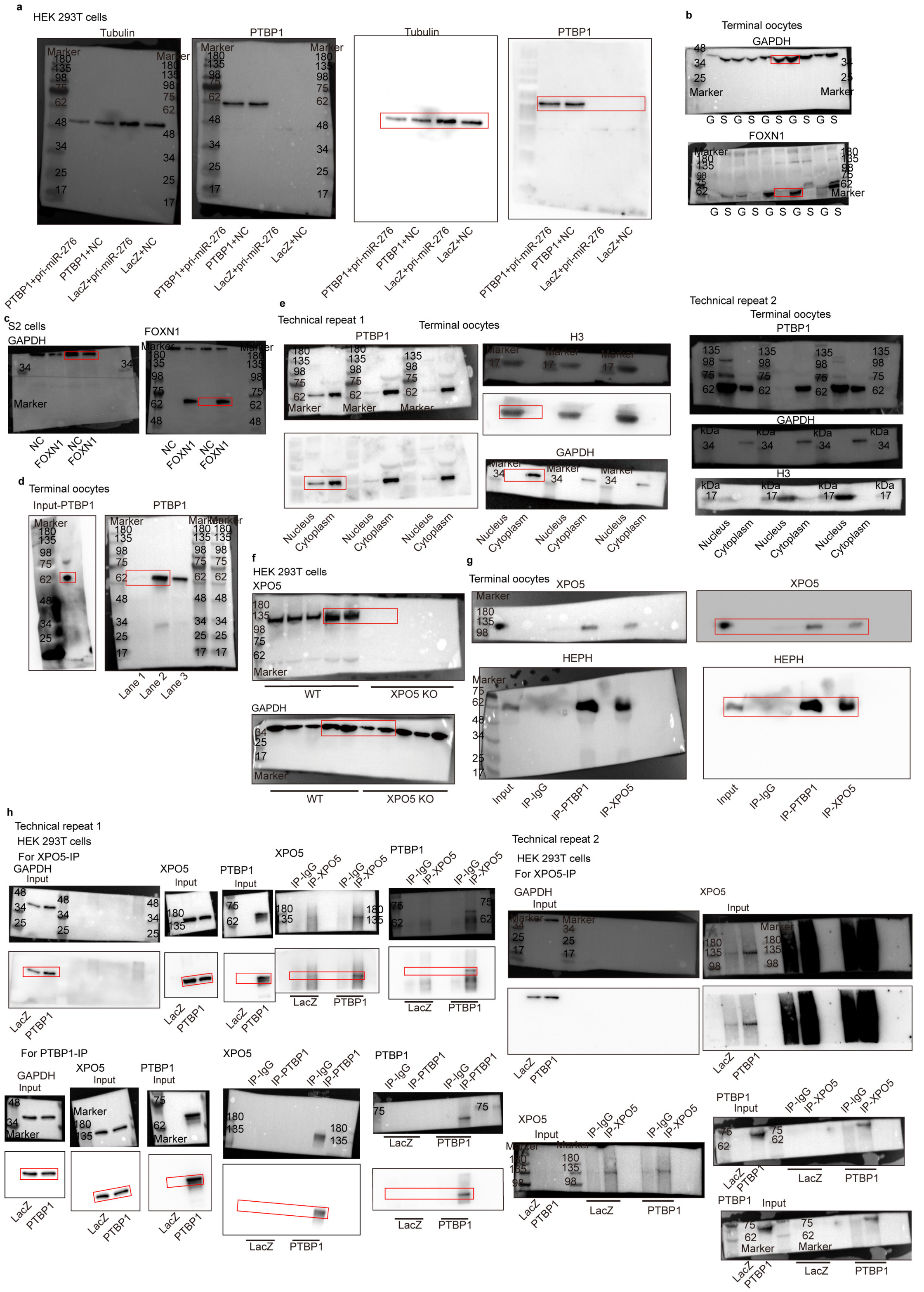

Supplementary Fig. 17. The full-size Western blot scans corresponding to the main figures.

(**a**) The uncropped Western blots of PTBP1 and Tubulin in Fig. 2i. pri-miR-276: primary miR-276; NC: negative control for pri-miR-276. (**b**) The uncropped Western blots of FOXN1 and GAPDH in Fig. 3j. n = 4 and 5 biologically independent locusts for gregarious (G) and

solitarious (S) locusts, respectively. (**c**) The uncropped Western blots of FOXN1 and GAPDH in Fig. 3n. n = 2 biologically independent locusts. (**d**) The uncropped Western blots of PTBP1 in input and RNA-pull down samples in Fig. 4a. Lane 2 and Lane 3 indicate two technical repeats of Western blots of PTBP1 from the protein samples in RNA pull-down assay. (**e**) The uncropped Western blots of PTBP1, GAPDH, and H3 in Fig. 4d. n = 3 biologically independent locusts. The experiments were performed twice and the uncropped Western blots are shown. (**f**) The uncropped Western blots of XPO5 and GAPDH in Fig. 4f. n = 5 biologically independent locusts. WT: wild type cells; KO; knockout cells. (**g**) The uncropped Western blots of XPO5 and PTBP1 in Fig. 4i. (**h**) The uncropped Western blots of XPO5, PTBP1, and GAPDH in Fig. 4l. The experiments were performed twice and the uncropped Western blots are shown. The bands that were cropped and presented in the main figures are indicated in red frames.

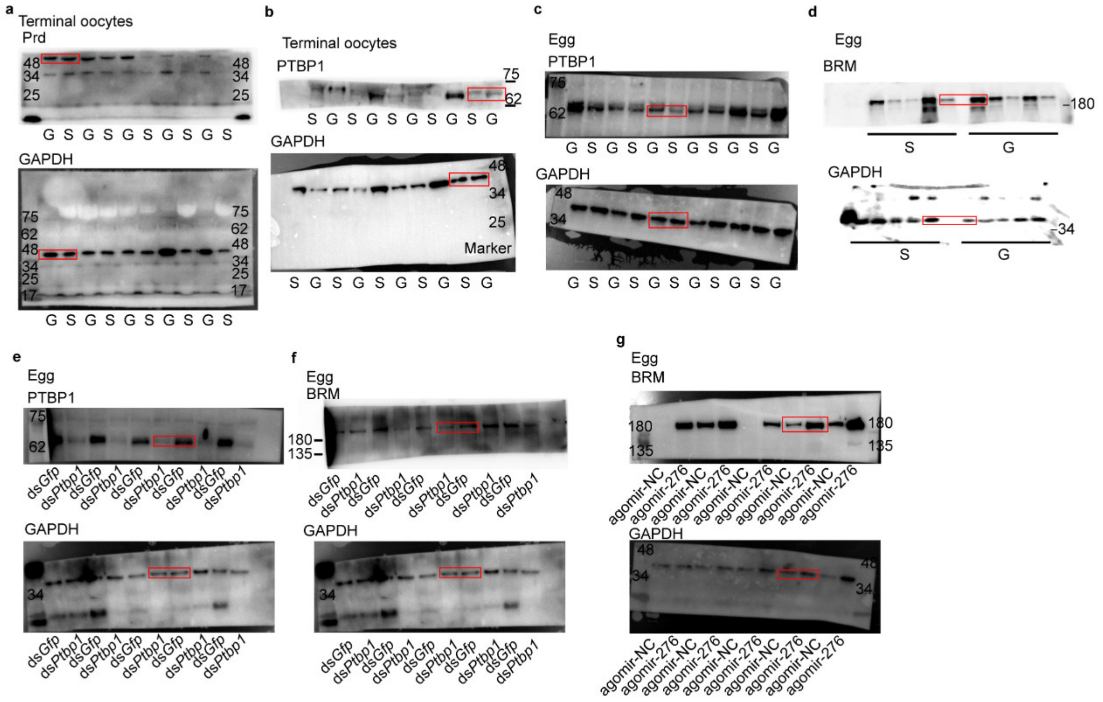

Supplementary Fig. 18. The full-size Western blot scans corresponding to Supplementary Figures. (**a**) The uncropped Western blot of Prd and GAPDH in Supplementary Fig. 6b. *n* = 5 biological repeats. (**b-c**) The uncropped Western blots of PTBP1 and GAPDH in Supplementary Fig. 14a and Supplementary Fig. 14b. *n* = 5-6 biologically independent locusts. (**d**) The uncropped Western blots of BRM and GAPDH in Supplementary Fig. 14d. *n* = 5 biological repeats. (**e**) The uncropped Western blots of PTBP1 and GAPDH in Supplementary Fig. 14e. *n* = 4 and 5 biologically independent locusts for ds*Gfp* and ds*Ptbp1*, respectively. Due to the disturbances caused by protein marker, the densitometry of the band in the second lane labeled as "ds*Gfp*" was not analyzed. (**f**) The uncropped Western blots of BRM and GAPDH in Supplementary Fig. 14g. *n* = 4 and 5 biologically independent locusts for ds*Gfp* and ds*Ptbp1*, respectively. Due to disturbances caused by protein marker, the densitometry of the band in the second lane labeled as "ds*Gfp*" was not analyzed. (**g**) The uncropped Western blots of BRM and GAPDH in Supplementary Fig. 14i. *n* = 5 biologically independent locusts. G: gregarious locusts; S: solitarious locusts. The bands that were cropped and presented in the Supplementary figures are indicated in red frames.

Supplementary Tables

Supplementary Table 1. The list of proteins that specifically pulled down by pre-miR-276.

Protein accession	Function annotation	Symbol
LOCMI10605	Exosome-mediated RNA decay	TTC37
LOCMI05182	Leucine-tRNA ligase	LARS
LOCMI06244	Regulator of nonsense transcripts 1	UPF1
LOCMI16762	ATP-dependent RNA helicase	DDX17
LOCMI11736	Cytoplasmic polyadenylation element-binding	CPEB1
LOCMI15806	Trifunctional enzyme subunit alpha	HADHA
LOCMI15119	Transcriptional repressor of the myelin basic protein gene	MYEF2
LOCMI04258	Polypyrimidine tract-binding protein 1	PTBP1
LOCMI10275	Polyribosome-associated RNA-binding protein	FMR1
LOCMI09143	AU-rich element binding protein	ELAVL3
LOCMI16664	RISC-loading complex subunit	TARBP2
LOCMI07033	CUGBP Elav-like family member 1	CELF1

Supplementary Table 2. The analysis results of hatching synchrony within egg-pods.

Treatment	Standard deviation (SD)	*n*	Levene' s test (one-tailed)	F-test (one-tailed)
G	0.16	220	$F = 43.60$	$F = 11.17$
S	0.54	195	$P = 1.24E-10$	$P < 0.0001$
Antagomir-NC	0.16	769	$F = 64.37$	$F = 5.10$
Antagomir-276	0.35	357	$P = 3.54E-15$	$P < 0.0001$
Agomir-NC	0.24	1,025	$F = 13.39$	$F = 2.01$
Agomir-276	0.17	554	$P = 0.00026$	$P < 0.0001$
ds*Gfp*	0.32	1,041	$F = 69.75$	$F = 2.25$
ds*Ptbp1*	0.49	769	$P = 1.32E-16$	$P < 0.0001$
ds*Ptbp1* + agomir-NC	0.55	403	$F = 14.35$	$F = 2.60$
ds*Ptbp1* + agomir-276	0.34	243	$P = 0.00017$	$P < 0.0001$

n indicates the number of biologically independent eggs.
