# Supplementary material for: Parental experiences orchestrate locust egg hatching synchrony by regulating nuclear export of precursor miRNA
Source: Nat Commun. 2024 May 21;15:4328. doi: 10.1038/s41467-024-48658-7 (PMC11109280; doi:10.1038/s41467-024-48658-7)
Supplement: Supplementary file 3 — Description of Additional Supplementary Files [file 41467_2024_48658_MOESM3_ESM.pdf]

Description of Additional Supplementary Files

File Name: Supplementary Data 1
Description: Primer sequences used for PCR amplification, qPCR, dsRNA synthesis and gRNA for gene knock out

File Name: Supplementary Data 2
Description: Probe sequences used for in situ fluorescence hybridization, RNA pull-down and EMSA

File Name: Supplementary Data 3
Description: Company names and catalog numbers for commercial reagents
