# Supplementary material for: Parental experiences orchestrate locust egg hatching synchrony by regulating nuclear export of precursor miRNA
Source: Nat Commun. 2024 May 21;15:4328. doi: 10.1038/s41467-024-48658-7 (PMC11109280; doi:10.1038/s41467-024-48658-7)
Supplement: Supplementary file 7 — Reporting Summary [file 41467_2024_48658_MOESM7_ESM.pdf]

nature portfolio

Corresponding author(s): Le Kang

Last updated by author(s): Apr 8, 2024

Reporting Summary

Nature Portfolio wishes to improve the reproducibility of the work that we publish. This form provides structure for consistency and transparency in reporting. For further information on Nature Portfolio policies, see our Editorial Policies and the Editorial Policy Checklist.

Statistics

For all statistical analyses, confirm that the following items are present in the figure legend, table legend, main text, or Methods section.

n/a	Confirmed	
☐	☒	The exact sample size (*n*) for each experimental group/condition, given as a discrete number and unit of measurement
☐	☒	A statement on whether measurements were taken from distinct samples or whether the same sample was measured repeatedly
☐	☒	The statistical test(s) used AND whether they are one- or two-sided<br>*Only common tests should be described solely by name; describe more complex techniques in the Methods section.*
☒	☐	A description of all covariates tested
☐	☒	A description of any assumptions or corrections, such as tests of normality and adjustment for multiple comparisons
☐	☒	A full description of the statistical parameters including central tendency (e.g. means) or other basic estimates (e.g. regression coefficient) AND variation (e.g. standard deviation) or associated estimates of uncertainty (e.g. confidence intervals)
☐	☒	For null hypothesis testing, the test statistic (e.g. *F*, *t*, *r*) with confidence intervals, effect sizes, degrees of freedom and *P* value noted<br>*Give P values as exact values whenever suitable.*
☒	☐	For Bayesian analysis, information on the choice of priors and Markov chain Monte Carlo settings
☒	☐	For hierarchical and complex designs, identification of the appropriate level for tests and full reporting of outcomes
☒	☐	Estimates of effect sizes (e.g. Cohen's *d*, Pearson's *r*), indicating how they were calculated

Our web collection on statistics for biologists contains articles on many of the points above.

Software and code

Policy information about availability of computer code

Data collection	Inbuilt software associated with the following equipments:<br>qPCR: qPCR data were generated by a Roche LightCycler 480 instrument. Gene expression level was calculated via 2^-ΔCt. In two-group comparisons, Student’s t test and Mann-Whitney U test were applied for analyzing the data with normal distribution and non-normal distribution, respectively. Kolmogorov-Smirnov test was used for normality test. All statistical results are in the “Source Data”. One-way ANOVA followed by Tukey’s multiple comparisons test was used for multigroup comparisons.<br>Confocal: LSM 710 confocal fluorescence microscope (Zeiss) equipped with ZEN 2012 software.<br>Mass spectrometry (MS/MS) analysis was performed at BPI (China).<br>EMSA and Western blot: Tanon-5200 Chemiluminescent Imaging System.<br>Dual-luciferase assay: GloMax 96 Microplate Luminometer.
Data analysis	Sequence alignment was performed with BioEdit software.<br>Primer Premier 6 was used for designing gene primer.<br>The proteins obtained from MS/MS were further analyzed with protein domain enrichment methods implemented in the LocustMine platform (Yang et al., 2019, Protein Cell 10, 883-901).<br>InterEvDock2 was used for prediction of interactions of proteins.<br>MatInspector program was used for predicting the transcription factors.<br>ImageJ software was used for measuring an analyzing the fluorescence intensities and Western band densities.<br>All statistical analyses were performed with GraphPad Prism 8 software.

For manuscripts utilizing custom algorithms or software that are central to the research but not yet described in published literature, software must be made available to editors and reviewers. We strongly encourage code deposition in a community repository (e.g. GitHub). See the Nature Portfolio guidelines for submitting code & software for further information.

Data

Policy information about availability of data

All manuscripts must include a data availability statement. This statement should provide the following information, where applicable:

- Accession codes, unique identifiers, or web links for publicly available datasets
- A description of any restrictions on data availability
- For clinical datasets or third party data, please ensure that the statement adheres to our policy

The published reference genome of migratory locust used for mapping is available at LocustBase [http://159.226.67.243]. The raw data for mass spectrometry have been deposited at iProX Consortium (IPX0006309000/PXD041810). Reviewer login information to access the data: https://proteomecentral.proteomexchange.org/cgi/GetDataset?ID=PXD041810. The miRNA data is available at miRbase database: https://www.mirbase.org/. The authors declare that the data supporting the findings of this study are available within the paper and its Supplementary Information.

Research involving human participants, their data, or biological material

Policy information about studies with human participants or human data. See also policy information about sex, gender (identity/presentation), and sexual orientation and race, ethnicity and racism.

Reporting on sex and gender	n/a
Reporting on race, ethnicity, or other socially relevant groupings	n/a
Population characteristics	n/a
Recruitment	n/a
Ethics oversight	n/a

Note that full information on the approval of the study protocol must also be provided in the manuscript.

Field-specific reporting

Please select the one below that is the best fit for your research. If you are not sure, read the appropriate sections before making your selection.

☒ Life sciences ☐ Behavioural & social sciences ☐ Ecological, evolutionary & environmental sciences

For a reference copy of the document with all sections, see nature.com/documents/nr-reporting-summary-flat.pdf

Life sciences study design

All studies must disclose on these points even when the disclosure is negative.

Sample size	The sample size for each experiment is indicated in the figure legends or the corresponding methods. Sample sizes were selected based on our previous experience to obtain statistical significance and reproducibility (He et al., 2016, Proc Natl Acad Sci USA 113, 584-589; Guo et al., 2018, Nat Commun 9, 1193; Zhang et al., 2020, PLoS Genet 16, e1008771; He et al., 2022, Cell Rep 39, 110593)
Data exclusions	No data were excluded
Replication	Quantitative experiments were performed with 4-6 biological replicates were applied in this studies. Some important assays were repeated for 3 times for each sample. All attempts to repeat the experiment were successful.
Randomization	The samples were allocated randomly into experimental groups.
Blinding	The investigators were blinded to group allocation during data collection and data analysis.

Reporting for specific materials, systems and methods

We require information from authors about some types of materials, experimental systems and methods used in many studies. Here, indicate whether each material, system or method listed is relevant to your study. If you are not sure if a list item applies to your research, read the appropriate section before selecting a response.

Materials & experimental systems

n/a	Involved in the study
☐	☒ Antibodies
☐	☒ Eukaryotic cell lines
☒	☐ Palaeontology and archaeology
☐	☒ Animals and other organisms
☒	☐ Clinical data
☒	☐ Dual use research of concern
☒	☐ Plants

Methods

n/a	Involved in the study
☒	☐ ChIP-seq
☒	☐ Flow cytometry
☒	☐ MRI-based neuroimaging

Antibodies

Antibodies used

Anti-PTBP1 antibody (rabbit) and anti-BRM antibody (mouse) are custom-made and purified by Abclonal Company (China; 1:500)
Anti-XPO5 antibody for locust (1:500 dilution; Abclonal, Cat# A3813, LOT 4000000838, CloneNo. ARC0838)
Anti-XPO5 antibody for HEK 293T cells (1:500 dilution; Abcam, Cat# ab57491, LOT GR3316680-10, CloneNo. 2C5-1B3)
Anti-FOXN1 antibody (Proteintech, Cat# 66337-1-Ig, LOT 10003426, CloneNo. 1D8B12)
Anti-Prd antibody (Proteintech, Cat#51036-2-AP)
Anti-GAPDH antibody (1:5000 dilution)
Anti-H3 antibody (1:5000 dilution, EASYBIO, Cat# BE3015, LOT 80780510)
Anti-Tubulin antibody (1:5000 dilution, EASYBIO, Cat# BE0025,LOT 80801222)
Anti-rabbit IgG secondary antibody (1:5000 dilution, Cat# BE0101, EASYBIO, LOT 80870103)
Anti-mouse IgG secondary antibody (1:5000 dilution, Cat# BE0102, EASYBIO, LOT 80790315)
Alexa Fluor 488 goat anti-rabbit IgG (1:5000 dilution, Cat# A11034, Life Technology, LOT 2286890)
Alexa Fluor 546 goat anti-mouse IgG (1:5000 dilution, Cat# A11003, Life Technology, LOT 2494860)

Validation

The antibodies of anti-PTBP1 (rabbit), anti-XPO5 (Abclonal, rabbit) and anti-Prd (rabbit) were validated in locusts by RNAi assay in this study (Supplementary Fig. 15).
The antibody against GAPDH (rabbit) was validated and applied in previous paper (Wang et al., 2013, Plos Pathog 9, e1003102).
The antibody against BRM (mouse) was validated and applied in previous paper (He et al., 2016, Proc Natl Acad Sci USA 113, 584-589).
Anti-H3 antibody (mouse, applications: WB, IHC, IP, IF)
http://www.bioeasytech.com/product/2358.html?goods_id=4246
Sun et al. Context-dependent tumor-suppressive BMP signaling in diffuse intrinsic pontine glioma regulates stemness through epigenetic regulation of CXXC5. Nature Cancer.2022(8)

anti-Tubulin antibody (mouse, applications: WB, IHC)
http://www.bioeasytech.com/product/2380.html?goods_id=4268
Ji et al. Slc6a8-Mediated Creatine Uptake and Accumulation Reprogram Macrophage Polarization via Regulating Cytokine Responses. IMMUNITY. 2019(8)

anti-XPO5 antibody (rabbit, applications: WB, IHC-P, ELISA)
https://abclonal.com.cn/catalog/A3813

anti-XPO5 antibody (mouse, application: WB, Flow Cyt, IP, ICC/IF, IHC-P)
https://www.abcam.cn/products/primary-antibodies/exportin-5-antibody-2c5-1b3-ab57491.html
Tseng CF et al. Transcriptional suppression of Dicer by HOXB-AS3/EZH2 complex dictates sorafenib resistance and cancer stemness. Cancer Sci 113:1601-1612 (2022).

anti-FOXN1 (mouse, application: FC, WB, ELISA),
https://www.ptgcn.com/products/FOXN1-Antibody-66337-1-Ig.htm

anti-Prd (rabbit, application: IHC, WB, ELISA)
https://www.ptgcn.com/products/PAX3-Antibody-51036-2-AP.htm#publications
Li et al.N-3 polyunsaturated fatty acids effectively protect against neural tube defects in diabetic mice induced by streptozotocin. Food Funct 2021, 12(19)

Eukaryotic cell lines

Policy information about cell lines and Sex and Gender in Research

Cell line source(s)	The S2 cell line and HEK 293T cell line used in this study were bought from ATCC.
Authentication	Morphology of each cell line was assessed by microscopy. HEK293 cells were identified by the vendor: https://www.atcc.org/products/crl-11268 (karyotyping). S2 cells were identified by the vendor: https://www.atcc.org/products/crl-1963.
Mycoplasma contamination	Not tested for mycoplasma contamination

Commonly misidentified lines (See ICLAC register)	No commonly misidentified cell lines were used in this study.

Animals and other research organisms

Policy information about studies involving animals; ARRIVE guidelines recommended for reporting animal research, and Sex and Gender in Research

Laboratory animals	The gregarious and solitary locusts (Locusta migratoria) used in the experiments were maintained in the Institute of Zoology, Chinese Academy of Sciences, Beijing, China. Well-ventilated cages (25 cm × 25 cm × 25 cm) were used to feed gregarious locusts at a density of approximately 300–400 insects per cage. Solitarious locusts were reared individually in separate and well-ventilated metal boxes with a volume of 10 cm × 10 cm × 25 cm. Both gregarious and solitarious locusts were reared under a photoperiod of 14 h/10 h light/dark at a temperature of 30 ± 2 degree centigrade. They were fed fresh greenhouse-grown wheat seedlings and wheat bran every day.
Wild animals	The study did not involve wild animals.
Reporting on sex	The locusts used for terminal oocytes sampling in this study were gregarious and solitarious females with the same length (~4.5 mm) of terminal oocytes.
Field-collected samples	The study did not involve samples collected from the field.
Ethics oversight	No ethical oversight was required as no vertebrate animals were involved in the study, and no ethical oversight of the experiments was required by our institution.

Note that full information on the approval of the study protocol must also be provided in the manuscript.
